# An Unexpected Case of Lisinopril-Associated Severe Hyponatremia

**DOI:** 10.7759/cureus.9039

**Published:** 2020-07-07

**Authors:** Yiran Jiang, Wenjing Cai, Meghane E Masquelin, Kaitlin Gordon

**Affiliations:** 1 Internal Medicine, University of Louisville School of Medicine, Louisville, USA; 2 Internal Medicine, University of Louisville, Louisville, USA; 3 Family Medicine, University of Louisville School of Medicine, Louisville, USA

**Keywords:** ace inhibitors, hyponatremia, lisinopril, siadh

## Abstract

Angiotensin-converting enzyme (ACE) inhibitors are a class of medications that have formed the backbone of hypertension management. Of these medications, lisinopril is one of the most commonly used. While known serious side effects of all ACE inhibitors include angioedema and hyperkalemia, ACE inhibitor-associated hyponatremia has been rarely reported. We present a patient with severe hyponatremia associated with lisinopril use and discuss the link between hyponatremia and ACE inhibitors.

## Introduction

Angiotensin-converting enzyme (ACE) inhibitors are a class of medications that inhibit the renin-angiontensin-aldosterone (RAS) system to facilitate a cascade of effects. Of these effects, the antihypertensive properties are perhaps the most well known. Despite the undisputed benefits to cardiovascular and renal health, ACE inhibitors have numerous drawbacks. In addition to hyperkalemia, angioedema, and teratogenicity, there have been reports postulating a link between hyponatremia and ACE inhibitor use. Lisinopril, one of cheapest and widely used ACE inhibitors since its medical approval in 1987, has also been implicated in these cases [1]. While the precise mechanism is unknown, the severity of the degree of hyponatremia experienced in certain patients warrants a closer look. Here, we present a patient who was admitted for angioedema and critical hyponatremia believed to be associated with lisinopril use and discuss the potential link and past literature.

## Case presentation

A 66-year-old African American male with a past medical history of hypertension, alcohol use, and asthma presented with facial swelling. He was discharged two months prior for a similar event related to lisinopril. On initial presentation, the patient's vitals were significant for heart rate of 104 beats per minute, respiratory rate of 30 breaths per minute, blood pressure of 190/100 mmHg, and oxygen saturation of 99% with a non-rebreather mask at 15 liters. Physical exam was significant for moderate to severe respiratory distress, inspiratory stridor, and subcostal and intercostal retractions. Lungs were clear to auscultation. The patient was otherwise alert and oriented. Arterial blood gas showed pH 7.447, pCO_2_ 39, pO_2_ 197, and HCO_3_ 27 on 60% FiO_2_. His initial metabolic panel was significant for sodium 104 mmol/L, potassium 2.6 mmol/L, chloride 66 mmol/L, blood urea nitrogen (BUN) 5 mg/dL (baseline 20 mg/dL), and creatinine 0.6 mg/dL (baseline 1.0 mg/dL). Glucose was 165 mg/dL. Magnesium and phosphorus were 1.1 and 2.1 mg/dL, respectively. Serum uric acid was low at 2.7 mg/dL. Complete blood count showed no leukocytosis or anemia. Urinalysis had a specific gravity of 1.010, moderate blood, and 15-29 red blood cells. Urine electrolytes were notable for a sodium of 48 mmol/L and osmolality of 217 mOsm/kg. Serum osmolality was 215 mOsm/kg. Urine and serum toxicology were negative for any substances. Alcohol level was negative.

**Figure 1:**
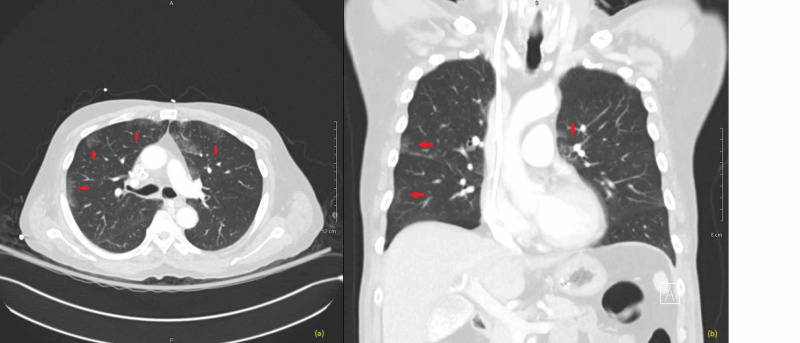
CT chest scan with contrast (a) Axial view of chest CT showing bilateral nonspecific ground-glass airspace opacities without evidence of malignancy. (b) Coronal view of chest CT at intersecting level redemonstrating scattered bilateral groundglass opacities.

Due to concern for airway protection, the patient was intubated and admitted to the intensive care unit. Initial chest x-ray held concern for infectious process, but antibiotics were stopped after infectious workup was negative. The patient was given two units of fresh-frozen plasma, famotidine, and Benadryl, and started on methylprednisolone 60 mg every six hours for angioedema. Lisinopril was held at this time. For his hyponatremia, the patient's labs were suspicious for syndrome of inappropriate antidiuretic hormone (SIADH) and a one-liter fluid restriction was initiated with nephrology consult. Thyroid and adrenal workup to rule out other causes of SIADH was unremarkable. A CT chest scan was done to evaluate for malignancy as a cause of SIADH, but did not demonstrate overt suspicious lung pathology (Figure 1). The patient's hyponatremia appropriately corrected over the course of one week and was 130 mmol/L upon discharge. Lisinopril was discontinued and marked as an allergy for the patient. On follow-up approximately nine months later, the patient's sodium had corrected and remained within normal limits.

## Discussion

The causes of hyponatremia vary greatly from medication-induced to dehydration, and the treatment is dependent on the cause. In our patient, the believed physiologic cause was an inappropriate secretion of antidiuretic hormone or SIADH. SIADH is characterized by the excessive release of antidiuretic hormone, a process that results in concentrated urine and increased water retention causing dilutional hyponatremia [2]. Laboratory studies often show a high urine sodium and a low serum uric acid. While the etiology ranges from central nervous system (CNS) disturbances to infections, one often seen offender includes medications. Typical causes include thiazides, antipsychotics, non-steroidal anti-inflammatories, and antidepressants. Our patient was notable for not being on any medications known to commonly cause hyponatremia.

Rarely, the use of ACE inhibitors in various settings has been linked to SIADH-related hyponatremia. To date, there have been less than 25 published case reports of ACE inhibitor-related hyponatremia when searched on PubMed. Of these, the duration of ACE inhibitor use varied significantly, ranging from new initiation to chronic medications. Patients also frequently had various inciting events that complicated the picture. However, few, if any, of the events were typical causative factors for SIADH-related hyponatremia. These events included heart failure exacerbation, myocarditis, and perioperative increase in fluid intake [1,3]. Implicated ACE inhibitors included enalapril, lisinopril, captopril, ramipril, and cilazapril. Of these, enalapril was the most common [3]. The varying chronicity of medication use in some cases seems to suggest a mechanism more nuanced and indirect than a direct effect of ACE inhibitors on antidiuretic hormone production.

While the particular mechanism is unknown, it has been postulated that the answer lies in the complex way the RAS functions on the peripheral and central level [1]. The CNS has a locally expressed RAS whereby the conversion of angiotensin I to angiotensin II is crucial in the activation of the hypothalamus and the release of vasopressin or antidiuretic hormone [4,5]. The variations in chemical makeup of different ACE inhibitors may impact their ability to affect the endogenous CNS RAS and thereby produce different degrees of effect, i.e., release more or less antidiuretic hormone [6]. It should be noted that this is a hypothesis that likely requires further research at the pharmacokinetic level. As noted above, many of the reported cases appeared to have an inciting event lending credibility to the theory that a variability in CNS inhibition of angiotensin I conversion due to ACE inhibitors may alter or lower the threshold at which new events trigger SIADH and subsequent hyponatremia.

The cessation of medication along with the initiation of fluid restriction typically resulted in the normalization of hyponatremia without significant adverse effects [3]. In one case, the re-initiation of lisinopril during the hospital stay corresponded with a decrease in serum sodium concentration [7]. In general, the outcome remains positive. As noted in our patient, the cessation of lisinopril and fluid restriction led to the correction of sodium levels which remained normal on follow-up. While it should be acknowledged that chronic alcoholism can cause hyponatremia, our patient's alcohol use continued without an adverse effect on his serum sodium level. 

## Conclusions

This report contributes to the body of literature that further elucidates a rare, but dangerous complication associated with a frequently prescribed medication. As with prior reports, we cannot concretely conclude the reason behind ACE inhibitor-associated SIADH and hyponatremia. However, given the number of similar reports and the dangers of severe hyponatremia, clinicians should be aware of the potential for hyponatremia in patients on lisinopril or other ACE inhibitor therapy, especially in an inpatient setting when critical illness may further lower the threshold to trigger SIADH.
